# Workplace Policy Awareness, Training, and Supervisory Support as Correlates of Generative Artificial Intelligence Use Among Practicing Nurses

**DOI:** 10.1002/nop2.71000

**Published:** 2026-11-03

**Authors:** Gregory A. Carter

**Affiliations:** ^1^ Indiana University School of Nursing Bloomington Indiana USA

**Keywords:** artificial intelligence, clinical decision‐making, generative artificial intelligence, governance, health policy, large language models, nurses, nursing, patient safety, workforce

## Abstract

**Aim:**

To estimate the prevalence and frequency of generative artificial intelligence (AI) use among practicing nurses and to determine whether four workplace governance conditions were independently associated with use.

**Design:**

US‐wide nonprobability cross‐sectional survey.

**Methods:**

Licensed, practicing nurses in the United States (*N* = 1136) recruited through a commercial, US‐wide nonprobability online panel completed a self‐administered questionnaire. Any work‐related use in the previous 12 months was modelled using binary logistic regression, and the frequency of clinical use, measured with an unbounded habitual‐use item, was modelled using proportional‐odds ordinal regression. Both models included workplace policy awareness, training status (training receipt and interest among untrained respondents), supervisor support, and organizational support and adjusted for role, setting, education, experience, age, gender, and geographic region. Sensitivity analyses examined ordinal‐covariate coding, the proportional‐odds assumption, and support‐item specification.

**Results:**

In the previous 12 months, 742 nurses (65.3%) reported using a generative AI tool for a work‐related task, and 528 (46.5%) reported at least weekly use for clinical tasks on a separate habitual‐frequency item without a bounded recall period. The two estimates are not nested because the frequency item used a different reference frame. Each point of supervisor support was associated with higher odds of any use (aOR = 1.42, 95% CI [1.17, 1.72]) and of more frequent clinical use (aOR = 2.05, 95% CI [1.75, 2.40]), with parallel associations for organizational support (aOR = 1.43 and 1.60). Nurses who were untrained and uninterested in training had markedly lower odds of both behaviours than trained nurses (aOR = 0.16 and 0.24); this comparison combines training receipt and interest and is not an estimate of training effectiveness. Uncertainty about whether a workplace policy existed, rather than its absence, was associated with lower use (aOR = 0.62 and 0.65). No statistically significant independent associations were detected for education, experience, age, gender, setting, or region, although role was associated with use.

**Conclusion:**

In this US‐wide nonprobability sample, generative AI use was common and associated with visible workplace governance conditions and professional role. The cross‐sectional design supports correlational, not causal, conclusions.

**Implications for the Profession and/or Patient Care:**

Communicated policy, accessible training, and frontline supervisory support warrant evaluation as potential governance approaches; this study does not establish whether frequent use was safe, appropriate, or outcome‐improving.

**Impact:**

The study addressed the lack of national evidence linking workplace governance to nurses' reported use of generative AI. Use was common and associated with supervisor support, training status (receipt and interest among untrained respondents), policy awareness, and professional role. The findings will inform nurses, nurse managers, health system leaders, and policy bodies responsible for evaluating and governing these tools.

**Reporting Method:**

Reporting followed the STROBE guidelines for cross‐sectional studies.

**Patient or Public Contribution:**

No Patient or Public Contribution. This survey of practicing nurses did not involve patients or members of the public in its design, conduct, analysis, or the preparation of the manuscript.

## 1 Introduction

Artificial intelligence (AI) has moved rapidly from experimental deployment to routine clinical infrastructure, and nursing, as the largest segment of the health care workforce, sits at the sharpest point of that transition. The scale of integration is no longer speculative. Analysis of the 2023 American Hospital Association Annual Survey found that 65% of US hospitals used AI‐driven predictive models, most commonly to predict inpatient health trajectories, identify high‐risk outpatients, monitor health, and automate scheduling and billing, with 79% of adopting hospitals relying on models supplied by their electronic health record developer (Nong et al. 2025). Across care settings, these applications support patient monitoring, predictive risk scoring, clinical decision support, documentation, and resource allocation, with evidence that well‐targeted tools can improve diagnostic accuracy, streamline workflows, and reduce administrative burden (El Arab, Al Moosa, et al. 2025; Fernandes et al. 2026; Park et al. 2025). Ambient documentation systems concretely illustrate the workflow promise, with audit‐log analyses demonstrating measurable reductions in on‐shift documentation time (Preiksaitis et al. 2026).

Generative AI extends this trajectory in a qualitatively different direction. Unlike embedded clinical systems that organizations select and manage, general‐purpose tools such as ChatGPT, Claude, and Gemini are freely available to individual clinicians, who can apply them to clinical questions, patient education, and documentation tasks at their own discretion and largely outside institutional oversight. Evidence of this discretionary use is accumulating. In a national survey of more than 1000 UK general practitioners conducted 1 year after the public release of ChatGPT, 20% reported using generative AI tools in clinical practice, most commonly to generate documentation after patient visits and to suggest differential diagnoses, despite the absence of formal guidance and unclear workplace policies (Blease et al. 2024). Population‐level data from Brazil show a parallel pattern among nurses, who primarily use generative tools for research and communication tasks, with adoption stratified by institutional capacity (Portilho and Marin 2026).

## 2 Background

The same properties that make generative tools flexible can also make them hazardous when adoption outpaces governance, and the failure modes are quantifiable. In a structured evaluation of ChatGPT‐generated medical content, 47% of the references the model produced were fabricated, and a further 46% were authentic but inaccurate, leaving only 7% both authentic and accurate (Bhattacharyya et al. 2023). When four commercially available large language models were tested against clinical scenarios, every model reproduced debunked race‐based medical content, and responses were inconsistent across repeated queries (Omiye et al. 2023). Evaluation frameworks for detecting fabrication, omission, and bias in clinical text remain heterogeneous and unstandardized (Bednarczyk et al. 2026).

Clinicians themselves articulate similar concerns, including data privacy, legal liability, algorithmic bias, the erosion of human oversight (Bogdanffy et al. 2026), and ethical judgements concerning appropriate clinical use remain divided even among professionals familiar with these tools (Cherrez‐Ojeda et al. 2026). When nurses use general‐purpose AI tools without workplace policy, formal training, or supervisory support, each of these risks shifts from the institutional level to the individual clinician, who must assess accuracy, protect patient information, and bear accountability alone. The practical question facing health systems is therefore not whether nurses will use generative AI but under what governance conditions that use occurs.

The nursing literature has expanded quickly but has not yet answered that question. Systematic reviews of the field document that existing quantitative work is dominated by cross‐sectional studies of attitudes, literacy, readiness, and behavioural intention rather than actual use (El Arab, Alshakihs, et al. 2025), frequently conducted among nursing students rather than practicing clinicians (Almarwani 2026; Zhu et al. 2025), and much of the evidence on adoption determinants derives from single countries or single health systems outside the United States (Chen et al. 2026; Wu 2026). Reviewers of this literature explicitly call for research that links self‐reported attitudes to objective use behaviour (El Arab, Alshakihs, et al. 2025). Studies that do capture organizational context point in a consistent direction: digital leadership, facilitating conditions, and perceived organizational support appear to shape nurses' engagement with generative tools, with reports of limited training opportunities and substantial uncertainty about institutional expectations (Lansdowne et al. 2025).

Umbrella and scoping reviews converge on the same recommendation, identifying governance frameworks, standardized training, and implementation research as the field's most pressing needs (El Arab, Al Moosa, et al. 2025; Park et al. 2025). What remains absent is national US evidence linking these governance conditions, specifically workplace AI policy, formal training, and supervisory and organizational support, to nurses' actual generative AI use behaviour rather than to intention or attitude alone.

## 3 The Study

### 3.1 Aims and Objectives

The present study was developed to address that gap in nursing‐relevant evidence. Drawing on a US‐wide cross‐sectional survey of practicing nurses, we pursued two aims. The first was to estimate the prevalence of work‐related use of generative AI and the frequency of its use for clinical tasks in this nonprobability sample. The second was to determine whether workplace policy awareness, training status (receipt and interest among untrained respondents), supervisory support, and organizational support were associated with use after adjustment for role, setting, education, experience, age, gender, and geographic region. The study was designed to identify correlates and practice‐relevant governance targets, not to establish causality or safety outcomes.

## 4 Methods

### 4.1 Design

This study employed a cross‐sectional design to survey practicing nurses in the United States. The study followed the Strengthening the Reporting of Observational Studies in Epidemiology (STROBE) guideline for cross‐sectional studies (File S1).

### 4.2 Instrument, Validity, and Reliability

Participants completed a self‐administered online questionnaire hosted on the Qualtrics platform. The instrument was developed for this study from the clinical AI, patient safety, and nursing governance literature and was reviewed for face validity, clarity, and relevance before fielding. To help ensure high‐quality responses, the questionnaire included an embedded attention check, a directed‐response item instructing respondents to select a specified option, to identify inattentive responding. The measures used in the analytic models are described below. Analytic item wording, response options, coding, and available routing information are provided in File S2. The complete instrument is available from the corresponding author upon reasonable request, as described in the Data Availability Statement. Governance and behavioural constructs were assessed using single items, which support face validity and reduce respondent burden but do not permit estimation of internal consistency reliability; this constraint is addressed in the limitations section.

#### 4.2.1 Outcomes

Two behavioural outcomes were analysed separately. Any work‐related generative AI use was measured with a single item asking whether, in the past 12 months, the respondent had used a generative AI tool such as ChatGPT, Claude, or Gemini for a work‐related task (no, yes) and was modelled as a binary outcome. Frequency of generative AI use for clinical tasks was measured with a single item on a five‐point ordered scale (never, less than once a month, about once a month, about once a week, daily or almost daily) and was modelled as an ordinal outcome. The two items index distinct constructs, the breadth of any work‐related use and the intensity of clinical use, and were not combined. The items also differ in reference frame: the any‐use item is anchored to the previous 12 months, whereas the frequency item asks about habitual use without a bounded recall window. The resulting proportions are therefore not nested and are not directly comparable.

#### 4.2.2 Governance Predictors

Workplace AI policy awareness was measured by asking whether the respondent's workplace had a policy governing the use of AI tools in patient care (no, yes, do not know) and was entered as a nominal variable, with no policy as the reference category. AI training status was a three‐level variable that combined receipt of formal training with training interest among the untrained: trained, untrained but interested in training, and untrained and not interested. Because interest was assessed only among respondents who reported no formal training, this composite preserved all cases and avoided the structural missingness that a standalone interest item would introduce, with trained respondents serving as the reference category. This composite measures training receipt and interest among untrained respondents, not the causal effect of training. Supervisor support and organizational support were each measured with a five‐point Likert item ranging from strongly disagree to strongly agree and were entered as linear terms.

#### 4.2.3 Covariates

Education (below baccalaureate, comprising diploma and associate degree; baccalaureate; and graduate, comprising master's, Doctor of Nursing Practice, and Doctor of Philosophy), years of nursing experience (five ordered bands), and age (ordered bands) were entered as ordinal linear terms in the primary models. Gender (female as reference), nursing role (bedside as reference, with charge nurse, advanced practice nurse, manager or administrator, educator, and other), primary employment setting (hospital as reference, with outpatient or ambulatory, home health or hospice, and an other category collapsing surgical center, public health, school, and other settings), and US census region (South as reference) were entered as nominal variables. Because education, experience, age, supervisor support, and organizational support were ordinal, we conducted sensitivity analyses treating these variables categorically.

### 4.3 Sampling and Recruitment

Participants were recruited through the Centiment research panel, a commercial online panel that provides access to a national pool of respondents. Recruitment targeted licensed, practicing nurses across the United States, and eligibility was established through self‐report screening items administered before the substantive survey. Licensure and employment were not independently verified. Respondents received compensation through the panel vendor according to the vendor's standard incentive structure; the research team did not directly compensate participants and did not receive respondent‐level incentive values. The survey platform and panel process used unique response and panel identifiers to monitor duplicate submissions; no duplicate response or panel identifiers were present in the analytic export. No quotas, weighting, or post‐stratification were applied. Completed submissions were screened for consent, eligibility, completeness of model variables, survey duration, and the embedded attention check.

### 4.4 Population and Sample

The target population was nurses currently licensed and practicing in the United States. The export used for analysis comprised 1239 completed submissions. Eligibility and quality gates excluded 95 records: 6 respondents reported that they were not currently licensed in the United States, 4 were not currently employed in a nursing role, 51 did not provide, supervise, or support direct patient care in a clinical setting, and 34 did not pass or reach the embedded attention‐quality gate. Among the 1144 eligible and quality‐passed records, 8 respondents declined consent, yielding an analysable sample of 1136 nurses with complete data on all variables included in the analytic models.

### 4.5 Inclusion and Exclusion Criteria

Participants were eligible if they were currently licensed to practice as a nurse in the United States, were currently employed in a nursing role, and had provided, supervised, or supported direct patient care in a clinical setting. After excluding respondents who were ineligible, did not pass or reach the embedded attention‐quality gate, or declined consent, the analysable sample size was 1136.

### 4.6 Sample Size

Of the 1136 analysable respondents, 742 reported any work‐related use of generative AI, yielding 394 respondents in the smaller outcome category for the binary model. With 22 predictor parameters in the full model, this corresponded to 17.9 observations in the smaller outcome category per predictor parameter, above the conventional threshold of 10 (Peduzzi et al. 1996).

### 4.7 Data Sources and Collection

The analysis used the July 3, 2026 Qualtrics export. Included responses were recorded from June 5 to July 3, 2026. The reviewed analytic sample of 1136 was retained for this revision; later responses were not incorporated.

### 4.8 Data Analysis

Data analysis occurred in multiple phases. We first summarized sample characteristics, governance conditions, and both outcomes using frequencies and percentages for categorical variables and means and standard deviations for the two support measures. We cross‐tabulated work‐related generative AI use in the previous 12 months against clinical use frequency to evaluate the distinct outcome reference frames. We then modelled any work‐related generative AI use with binary logistic regression and frequency of clinical use with proportional‐odds ordinal logistic regression, entering the same set of governance predictors and covariates in both models. Unadjusted odds ratios and adjusted odds ratios (aORs) are reported with 95% confidence intervals.

The proportional‐odds assumption was evaluated using the Brant test (Brant 1990), using the brant package (Schlegel and Steenbergen 2020); for a five‐level outcome and 22 predictor terms, the omnibus test has 66 degrees of freedom. When a term violated the assumption, we retained the primary model and examined cumulative‐logit threshold models that retained age as a sensitivity check on the governance estimates. Analyses were conducted in IBM Statistical Package for the Social Sciences version 31 (IBM Corp 2025), except the Brant test and sensitivity checks, which were computed in R version 4.6.1 (R Core Team 2026), with statistical significance set at *p* < 0.05 (two‐tailed). Because two multivariable models with multiple covariates were estimated, secondary covariate findings with borderline *p*‐values were interpreted with caution.

### 4.9 Ethical Considerations

Participants were required to review a study information sheet and provide informed consent before proceeding. Respondents who declined consent were excluded from the analysable sample. All study materials and procedures were approved by the blinded Institutional Review Board (protocol #31973). Participation was voluntary, responses were collected anonymously, and no personally identifying information was retained in the analytic dataset.

## 5 Results

### 5.1 Subjects

The analysable sample comprised 1136 nurses (Table 1). Most were female (81.5%) and baccalaureate‐prepared (50.3%), with an additional 19.1% holding a graduate degree. Respondents were distributed across the experience spectrum, from early‐career to more than 20 years in practice and worked predominantly in hospital settings (63.4%). Compared with the 2024 National Nursing Workforce Survey, this sample had a lower proportion of women (81.5% vs. 89.2%), a similar proportion with baccalaureate or higher education (69.4% vs. 72.9%), a higher hospital‐setting share (63.4% vs. 53.3%), and higher proportions of advanced practice nurses (19.3% vs. 10.8%) and managers or administrators (15.9% vs. 7.3%). These differences support interpreting prevalence estimates as sample estimates rather than weighted national workforce estimates.

**TABLE 1:** Sample characteristics and governance conditions (*N* = 1136).

Characteristic	*n*	%
Highest nursing education		
Diploma	105	9.2
Associate degree	243	21.4
Baccalaureate	571	50.3
Master's	159	14.0
Doctor of Nursing Practice	50	4.4
Doctor of Philosophy	8	0.7
Years of nursing experience		
Less than 1 year	13	1.1
1 to 5 years	273	24.0
6 to 10 years	293	25.8
11 to 20 years	322	28.3
More than 20 years	235	20.7
Age (years)		
18 to 24	23	2.0
25 to 34	311	27.4
35 to 44	380	33.5
45 to 54	266	23.4
55 to 64	150	13.2
65 to 74	6	0.5
Gender		
Female	926	81.5
Male	210	18.5
Nursing role		
Bedside	385	33.9
Charge nurse	266	23.4
Advanced practice nurse	219	19.3
Manager or administrator	181	15.9
Educator	23	2.0
Other	62	5.5
Primary employment setting		
Hospital	720	63.4
Outpatient or ambulatory	143	12.6
Home health or hospice	141	12.4
Public health	35	3.1
Surgical center	20	1.8
School	9	0.8
Other	68	6.0
United States Census Region		
South	426	37.5
Midwest	277	24.4
Northeast	247	21.7
West	161	14.2
US territory	25	2.2
Workplace AI policy		
Yes	534	47.0
No	369	32.5
Do not know	233	20.5
AI training status		
Trained	572	50.4
Untrained, interested	359	31.6
Untrained, not interested	205	18.0
Workplace support, *M* (SD)		
Supervisor support	3.42 (1.20)	
Organizational support	3.42 (1.25)	

*Note:* Percentages are column percentages of the analysable sample. *M* and SD are reported for the two support items, each rated on a five‐point scale (1 = strongly disagree, 5 = strongly agree).

Governance conditions varied widely. Just under half of respondents (47.0%) reported that their workplace had a policy governing AI use in patient care, while 32.5% reported no policy and 20.5% were unsure. Half (50.4%) had received formal training on AI tools; among those who had not, most expressed interest in training (31.6% of the full sample), while 18.0% were untrained and not interested. Supervisor support (*M* = 3.42, SD = 1.20) and organizational support (*M* = 3.42, SD = 1.25) were, on average, near the midpoint of the scale.

### 5.2 Work‐Related Use and Clinical‐Use Frequency

Generative AI use was common, but the two outcome proportions were not directly comparable. In the previous 12 months, 742 of the sample (65.3%) had used a generative AI tool for a work‐related task. Use for clinical tasks specifically was measured with a separate habitual‐frequency item: 839 respondents (73.9%) reported at least some clinical use, 528 (46.5%) used generative AI weekly or more often, and 265 (23.3%) reported daily or near‐daily clinical use. Cross‐tabulation showed that 40 respondents who reported no work‐related use in the previous 12 months nonetheless reported weekly or daily clinical use, and 129 reported at least some clinical use. This pattern is consistent with the different reference frames and precludes treating the responses as nested measures. Accordingly, the clinical‐frequency estimate is used as an intensity measure and is not interpreted as 12‐month clinical‐use prevalence.

### 5.3 Correlates of Any Work‐Related Use

The binary logistic model was significant overall (McFadden *R* ^2^ = 0.26; Brier score = 0.156; c statistic = 0.82), and governance support emerged as a robust correlate of use (Table 2). Each additional point of supervisor support was associated with 42% higher adjusted odds of any work‐related use (aOR = 1.42, 95% CI [1.17, 1.72], *p* < 0.001), and each point of organizational support with 43% higher adjusted odds (aOR = 1.43, 95% CI [1.18, 1.72], *p* < 0.001). The two support measures were correlated (*r* = 0.77), had nearly identical means, and did not differ statistically in paired comparison (mean difference = −0.01, *p* = 0.775). The paired comparison concerns the support ratings, not the regression coefficients. Both support items were associated with use; their odds ratios alone do not establish that one support source was stronger than the other.

**TABLE 2:** Binary logistic regression predicting any work‐related generative AI use in the previous 12 months.

Predictor	Unadjusted OR (95% CI)	*p*	Adjusted OR (95% CI)	*p*
Workplace AI policy (ref = no policy)				
Has policy	1.95 [1.46, 2.61]	< 0.001	0.72 [0.49, 1.07]	0.103
Do not know	0.47 [0.33, 0.65]	< 0.001	0.62 [0.42, 0.92]	0.019
AI training status (ref = trained)				
Untrained, interested	0.27 [0.20, 0.37]	< 0.001	0.62 [0.40, 0.94]	0.024
Untrained, not interested	0.06 [0.04, 0.08]	< 0.001	0.16 [0.09, 0.26]	< 0.001
Supervisor support	2.40 [2.11, 2.73]	< 0.001	1.42 [1.17, 1.72]	< 0.001
Organizational support	2.34 [2.08, 2.65]	< 0.001	1.43 [1.18, 1.72]	< 0.001
Nursing role (ref = bedside nurse)				
Charge nurse	2.37 [1.69, 3.32]	< 0.001	2.08 [1.38, 3.14]	< 0.001
Advanced practice nurse	2.95 [2.04, 4.28]	< 0.001	2.09 [1.29, 3.39]	0.003
Manager or administrator	2.17 [1.48, 3.16]	< 0.001	1.79 [1.13, 2.84]	0.013
Educator	1.39 [0.59, 3.30]	0.449	1.72 [0.61, 4.83]	0.303
Other role	1.24 [0.72, 2.14]	0.435	1.91 [0.99, 3.67]	0.052
Employment setting (ref = hospital)				
Outpatient or ambulatory	0.67 [0.47, 0.98]	0.037	0.93 [0.58, 1.48]	0.752
Home health or hospice	0.76 [0.53, 1.11]	0.161	0.88 [0.55, 1.40]	0.583
Other setting	0.62 [0.43, 0.91]	0.015	1.16 [0.72, 1.89]	0.538
Education	1.12 [0.94, 1.33]	0.224	0.88 [0.69, 1.12]	0.289
Years of nursing experience	0.81 [0.72, 0.90]	< 0.001	0.96 [0.80, 1.16]	0.682
Age	0.81 [0.72, 0.91]	< 0.001	0.88 [0.72, 1.06]	0.186
Male gender	1.37 [0.99, 1.90]	0.058	0.98 [0.66, 1.47]	0.939
Region (ref = South)				
Midwest	0.78 [0.57, 1.08]	0.134	0.89 [0.60, 1.31]	0.545
Northeast	0.88 [0.63, 1.23]	0.454	0.85 [0.57, 1.28]	0.445
West	0.92 [0.63, 1.36]	0.684	0.95 [0.60, 1.50]	0.810
U.S. territory	0.85 [0.37, 1.98]	0.709	1.17 [0.41, 3.37]	0.769

*Note:* Training status combines receipt of formal training and interest among untrained respondents; it does not estimate a causal training effect. Unadjusted estimates are from single‐predictor models. Adjusted estimates include all variables shown. Ordinal covariates (education, experience, age) and support items were entered as linear terms in the primary model; categorical sensitivity analyses supported the associations between support and use. Model McFadden *R* ^2^ = 0.26; Brier score = 0.156; c statistic = 0.82.

Abbreviations: aOR, adjusted odds ratio; CI, confidence interval; *N*, 1136; OR, odds ratio; ref, reference category.

The composite training‐status variable showed a pronounced gradient. Relative to trained nurses, those who were untrained and not interested in training had markedly lower odds of use (aOR = 0.16, 95% CI [0.09, 0.26], *p* < 0.001), and untrained but interested nurses had intermediate odds (aOR = 0.62, 95% CI [0.40, 0.94], *p* = 0.024). Nurses uncertain whether their workplace had an AI policy were less likely to use generative AI than those reporting no policy (aOR = 0.62, 95% CI [0.42, 0.92], *p* = 0.019), whereas reporting a policy did not differ significantly from reporting none (aOR = 0.72, 95% CI [0.49, 1.07], *p* = 0.103). Role was also associated with use: charge nurses (aOR = 2.08, 95% CI [1.38, 3.14], *p* < 0.001), advanced practice nurses (aOR = 2.09, 95% CI [1.29, 3.39], *p* = 0.003), and managers or administrators (aOR = 1.79, 95% CI [1.13, 2.84], *p* = 0.013) all had higher adjusted odds of any work‐related use than bedside nurses. Education, experience, age, gender, employment setting, and census region showed no statistically significant independent associations with use in this sample.

### 5.4 Correlates of Clinical‐Use Frequency

The omnibus Brant test did not detect a violation of the proportional‐odds assumption, *χ* ^2^(66) = 80.38, *p* = 0.110. The 66 degrees of freedom reflect a five‐category outcome and 22 predictor terms, with (5–2) × 22 tested constraints. A single covariate, age, violated the assumption (*p* = 0.004). Retaining age in cumulative‐threshold sensitivity models left the governance findings substantively unchanged: supervisor support and organizational support were positively associated across all four thresholds, and the untrained‐not‐interested group had lower odds across thresholds. The ordinal model had McFadden *R* ^2^ = 0.19.

Governance support was again a strong correlate (Table 3). Each additional point of supervisor support was associated with roughly twice the adjusted odds of more frequent clinical use (aOR = 2.05, 95% CI [1.75, 2.40], *p* < 0.001), and each point of organizational support with 60% higher adjusted odds (aOR = 1.60, 95% CI [1.37, 1.87], *p* < 0.001). In categorical sensitivity models, higher support categories generally showed a monotonic increase in use, supporting the associations between support and use without requiring a strict equal‐interval interpretation of the five‐point items.

**TABLE 3:** Proportional‐odds ordinal regression predicting habitual frequency of generative AI use for clinical tasks without a bounded recall period.

Predictor	Unadjusted OR (95% CI)	*p*	Adjusted OR (95% CI)	*p*
Workplace AI policy (ref = no policy)				
Has policy	2.19 [1.72, 2.78]	< 0.001	0.97 [0.73, 1.28]	0.813
Do not know	0.45 [0.34, 0.61]	< 0.001	0.65 [0.47, 0.90]	0.011
AI training status (ref = trained)				
Untrained, interested	0.27 [0.21, 0.35]	< 0.001	0.94 [0.68, 1.29]	0.686
Untrained, not interested	0.05 [0.03, 0.07]	< 0.001	0.24 [0.15, 0.36]	< 0.001
Supervisor support	3.39 [3.02, 3.81]	< 0.001	2.05 [1.75, 2.40]	< 0.001
Organizational support	3.03 [2.71, 3.38]	< 0.001	1.60 [1.37, 1.87]	< 0.001
Nursing role (ref = bedside nurse)				
Charge nurse	2.05 [1.55, 2.72]	< 0.001	1.45 [1.06, 1.98]	0.019
Advanced practice nurse	2.16 [1.61, 2.90]	< 0.001	1.27 [0.89, 1.79]	0.183
Manager or administrator	1.79 [1.30, 2.46]	< 0.001	1.22 [0.86, 1.74]	0.262
Educator	0.94 [0.45, 1.97]	0.869	0.66 [0.29, 1.50]	0.324
Other role	0.88 [0.54, 1.42]	0.592	1.06 [0.62, 1.83]	0.831
Employment setting (ref = hospital)				
Outpatient or ambulatory	0.68 [0.49, 0.94]	0.020	1.02 [0.71, 1.48]	0.897
Home health or hospice	0.80 [0.58, 1.11]	0.177	1.02 [0.71, 1.48]	0.906
Other setting	0.59 [0.43, 0.82]	0.002	1.09 [0.75, 1.59]	0.650
Education	1.13 [0.97, 1.31]	0.108	0.98 [0.82, 1.17]	0.808
Years of nursing experience	0.87 [0.80, 0.96]	0.006	0.93 [0.81, 1.08]	0.358
Age	0.91 [0.82, 1.00]	0.045	1.10 [0.95, 1.27]	0.225
Male gender	1.55 [1.18, 2.03]	0.002	1.21 [0.90, 1.63]	0.198
Region (ref = South)				
Midwest	0.82 [0.63, 1.08]	0.156	0.91 [0.68, 1.22]	0.547
Northeast	0.98 [0.74, 1.29]	0.864	0.95 [0.70, 1.28]	0.739
West	0.82 [0.59, 1.14]	0.231	0.85 [0.60, 1.20]	0.352
U.S. territory	0.83 [0.41, 1.66]	0.599	1.00 [0.45, 2.21]	0.990

*Note:* Training status combines receipt of formal training and interest among untrained respondents; it does not estimate a causal training effect. Unadjusted estimates are from single‐predictor proportional‐odds models. Adjusted estimates include all variables shown. Odds ratios greater than 1 indicate higher odds of more frequent clinical use. The omnibus Brant test did not detect a violation of the proportional‐odds assumption, *χ* ^2^(66) = 80.38, *p* = 0.110; ordinal‐model McFadden *R* ^2^ = 0.19.

Abbreviations: aOR, adjusted odds ratio; CI, confidence interval; *N*, 1136; OR, odds ratio; ref, reference category.

## 6 Discussion

### 6.1 Principal Findings

In this study of 1136 practicing US nurses recruited through a commercial nonprobability panel, generative AI use was common. Two‐thirds of respondents (65.3%) had used a generative AI tool for a work‐related task in the past year. A separate clinical‐frequency item indicated frequent clinical use, but because it did not use the same bounded recall period, it should be interpreted as an intensity measure rather than a nested prevalence estimate. Workplace governance conditions, including support and training status (receipt and interest among untrained respondents), were associated with both outcomes, while professional role also patterned use.

### 6.2 Comparison With Prior Work

The proportion reporting work‐related use in this sample was higher than the clinical‐use proportion reported in an earlier clinician survey, although the measures and samples differ. 1 year after the public release of ChatGPT, 20% of more than 1000 UK general practitioners reported using generative AI in clinical practice (Blease et al. 2024). Several differences complicate this comparison: the additional years of diffusion between the two surveys; a measure that captured any work‐related task rather than narrowly defined clinical use; occupational differences in task structure; and an online panel that may overrepresent digitally engaged nurses. These cross‐sectional surveys do not establish a temporal trend or a population‐level difference in adoption.

The estimates also extend the nursing literature, which systematic reviews characterize as dominated by cross‐sectional studies of attitudes, literacy, readiness, and intention, often in student samples (Almarwani 2026; El Arab, Alshakihs, et al. 2025; Zhu et al. 2025). Those reviews explicitly call for evidence linking self‐report constructs to use behaviour (El Arab, Alshakihs, et al. 2025). A review of nurses' experiences with AI in clinical practice also identifies training and facilitating conditions as relevant to implementation (Scott et al. 2026). The present study answers that call in part by supplying self‐reported behavioural estimates from a US‐wide nonprobability sample, while sharing the constraint that behaviour was self‐reported rather than observed.

The frequency findings provide context for the safety literature. Concerns about generative AI in medicine are typically illustrated with structured evaluations conducted outside of care delivery: in one such assessment, 47% of the references ChatGPT produced were fabricated outright, and a further 46% were authentic but inaccurate (Bhattacharyya et al. 2023), and all four commercial large language models tested against clinical scenarios reproduced debunked race‐based content (Omiye et al. 2023). In this US‐wide nonprobability sample, nearly half reported at least weekly clinical use on the unbounded habitual‐frequency item. This finding underscores the relevance of evaluating these risks in nursing practice, but the survey did not measure whether respondents encountered these failures or whether patients were exposed to harm.

Two features make the exposure difficult to manage. First, generative errors are often well articulated. A fabricated medication dose or frequency embedded in an otherwise accurate patient‐education document may appear authentic while hiding a clinically important error. Similarly, a discharge summary rewritten to simplify language may improve readability while introducing omissions or altered meaning. These examples underscore that the frequency of use should not be equated with safe or appropriate use.

The governance findings converge with and extend an emerging organizational literature. Supervisor support was associated with 42% higher adjusted odds of any work‐related use (aOR = 1.42) and roughly twice the adjusted odds of more frequent clinical use per scale point (aOR = 2.05). Organizational support was also independently associated with both outcomes (aOR = 1.43 and 1.60). The two support items were highly correlated. Their nearly identical means describe reported support levels and do not compare the regression coefficients. Both support sources were associated with use; these results do not establish the relative effectiveness of supervisory and organizational interventions.

The policy findings appear to address a gap in the literature we identified. Reporting that a workplace policy existed was not significantly associated with use relative to reporting no policy in either model (any use: aOR = 0.72, *p* = 0.103; clinical‐use frequency: aOR = 0.97, *p* = 0.813), whereas not knowing whether a policy existed was associated with lower odds of both any use (aOR = 0.62, *p* = 0.019) and more frequent clinical use (aOR = 0.65, *p* = 0.011). This pattern suggests that policy communication, not merely policy existence, warrants attention in future implementation research.

The training gradient identifies where governance exposure concentrates, but it should not be interpreted as a causal estimate of training effectiveness. Nurses who were untrained and uninterested in training had lower adjusted odds of reported use (any use: aOR = 0.16; frequency: aOR = 0.24). Another group comprised the 31.6% of the sample who were untrained but interested. Their intermediate odds of work‐related use and non‐significant adjusted association with clinical‐use frequency suggest that interest in training may index readiness, prior use, or unmet organizational support rather than training exposure alone.

Education, experience, age, gender, employment setting, and census region showed no statistically significant independent associations in the adjusted models, but professional role was associated with use. Charge nurses, advanced practice nurses, and managers or administrators had higher adjusted odds of any work‐related use than bedside nurses, and charge nurses also had higher adjusted odds of more frequent clinical use. Nonsignificant associations in this sample do not establish the absence of demographic differences in the broader nursing population.

### 6.3 Implications for Practice and Policy

These findings carry three implications for practice and policy. First, health systems could evaluate broadly accessible, role‐tailored training that addresses the actual failure modes of generative tools: verification of outputs against source material, boundaries around protected health information, recognition of fabricated citations and altered clinical meaning, and procedures for documenting and reporting concerns. Second, policy communication warrants evaluation as part of implementation; the study did not assess policy adherence. Third, frontline supervisors are plausible targets for future intervention to examine whether supervisory involvement supports questions, disclosure of uncertainty, and reporting of unsafe AI use. The training variable combines receipt and interest among untrained respondents. These are governance implications from cross‐sectional associations, not evidence that any specific intervention caused safer or more appropriate AI use.

### 6.4 Strengths and Limitations of the Work

All key measures were self‐reported, and several central constructs were measured with single items. The support items were treated as linear terms in the primary models, although categorical sensitivity analyses supported the associations between support and use. The clinical‐use frequency item did not include the same 12‐month recall period as the any‐use item, creating non‐nested estimates and requiring caution in interpretation. The cross‐sectional design precludes temporal or causal inference: nurses who use generative AI may become more aware of policies, seek training, or perceive support differently from non‐users. Finally, the study measured reported frequency of generative AI use, not whether use was safe, policy‐concordant, clinically appropriate, or associated with patient outcomes.

The study has several strengths worth discussing. It draws on a large US‐wide sample of practicing nurses rather than students; it models reported use behaviour rather than intention or attitude; it examines two complementary behavioural outcomes; and it reports sensitivity analyses for model specification and the proportional‐odds assumption. Several limitations temper interpretation. The national scope of recruitment does not make this a nationally representative probability sample. Recruitment through a nonprobability online panel may overrepresent digitally engaged nurses, potentially inflating reported use proportions and affecting observed associations. Eligibility was established through self‐report screening rather than independent licence verification, and no weighting or post‐stratification was applied. The sample included higher proportions of advanced practice nurses, managers or administrators, and hospital‐based respondents than the 2024 National Nursing Workforce Survey, which further supports cautious interpretation of prevalence estimates. The exclusion of respondents who failed the attention check, while improving data quality, may not be random with respect to the constructs studied.

### 6.5 Recommendations for Further Research

Several extensions follow from these findings. Longitudinal, prospective, and quasi‐experimental designs are needed to establish the direction of the supervisor‐use association and to test whether policy communication or training changes behaviour. The hypothesized psychological‐safety mechanism (Edmondson 1999) linking supervisory support to disclosure and error surfacing should be examined directly in mediation designs. Future instruments should replace single‐item governance measures with validated multi‐item scales, align the reference frames of behavioural outcome items, capture the task content of clinical use, and directly measure verification behaviours and safety outcomes. Probability‐based sampling and workforce weighting would strengthen the external validity of national prevalence estimates.

## 7 Conclusion

In this US‐wide nonprobability sample, reported use was common and associated with visible workplace governance conditions and professional role. The findings shift the relevant question from whether nurses are encountering generative AI to how health systems should study, communicate, train for, and govern the use already underway. Communicated policy, accessible training, and frontline leadership are plausible targets for future implementation and intervention research, but this cross‐sectional self‐report study cannot determine whether they cause greater, safer, or more appropriate use.

## Author Contributions

**Gregory A. Carter:** conceptualization, investigation, writing – original draft, methodology, validation, writing – review and editing, software, formal analysis, project administration.

## Funding

The author has nothing to report.

## Disclosure

Statistical guidelines statements: The author has checked to make sure that this submission conforms, as applicable, to the journal's statistical guidelines. (a) There is a statistician on the author team: Gregory A. Carter (sole author). (b) The author affirms that the methods used in the data analyses are suitably applied to the data within the study design and context, and that the statistical findings have been implemented and interpreted correctly. (c) The author agrees to take responsibility for ensuring that the choice of statistical approach is appropriate and is conducted and interpreted correctly. Reporting guideline: STROBE (Strengthening the Reporting of Observational Studies in Epidemiology). A completed STROBE checklist is provided as File S1.

## Ethics Statement

All study materials and procedures were approved by the Indiana University Institutional Review Board (protocol #31973).

## Consent

All participants reviewed a study information sheet and provided informed consent before proceeding. Responses were collected anonymously, and no personally identifying information was collected or retained in the analytic dataset.

## Conflicts of Interest

The author declares no conflicts of interest.

## Supporting information


**File S1:** STROBE statement: checklist of items for cross‐sectional studies.

**File S2:** Participant flow, analytic items, response options, coding, and routing documentation for the reviewed sample of 1136.

## Data Availability

De‐identified study data and the complete survey instrument are available from the corresponding author upon reasonable request. Public release of the participant‐level dataset is deferred while the author completes ongoing analyses. Researchers may contact the corresponding author using the details on the title page, describing the research purpose and the data or survey materials requested. Analytic item documentation is provided in File S2. Analysis syntax is supplied with the revision package and is also available upon reasonable request.
