# Supplementary material for: Workplace Policy Awareness, Training, and Supervisory Support as Correlates of Generative Artificial Intelligence Use Among Practicing Nurses
Source: Nurs Open. 2026 Nov 3;13(11):e71000. doi: 10.1002/nop2.71000 (PMC13731167; doi:10.1002/nop2.71000)
Supplement: Supplementary file 1 [file NOP2-13-e71000-s001.docx]

**Supplementary File 1**

**STROBE Statement: Checklist of Items for Cross-Sectional Studies**

Manuscript: Workplace Policy Awareness, Training, and Supervisory Support as Correlates of Generative Artificial Intelligence Use Among Practicing Nurses

Page and continuous line references refer to the locally rendered V8 clean manuscript (28 pages). Confirm pagination after journal upload conversion. Locations identify the corresponding reported content.

| **Item** | **Topic** | **Recommendation** | **Page(s)** |
| --- | --- | --- | --- |
|  | *Introduction* |  |  |
| 1 | **Title and abstract** | (a) Indicate the study design with a commonly used term in the title or abstract. (b) Provide in the abstract an informative and balanced summary of what was done and what was found. Addressed in Title and Abstract (Design: cross-sectional survey; structured abstract). | pp. 1–2, lines 2–45 |
|  | *Introduction* |  |  |
| 2 | **Background/rationale** | Explain the scientific background and rationale for the investigation. Addressed in Introduction and Background. | pp. 3–5, lines 50–112 |
| 3 | **Objectives** | State specific objectives, including any prespecified hypotheses. Addressed in 3.1 Aims and Objectives. | p. 6, lines 114–123 |
|  | *Methods* |  |  |
| 4 | **Study design** | Present key elements of study design early in the paper. Addressed in 4.1 Design. | p. 6, lines 125–128 |
| 5 | **Setting** | Describe the setting, locations, and relevant dates, including periods of recruitment and data collection. Addressed in 4.1 Design, 4.3 Sampling and Recruitment, and 4.7 Data Sources and Collection. | pp. 8–10, lines 175–211 |
| 6 | **Participants** | Give the eligibility criteria and the sources and methods of selection of participants. Addressed in 4.3 Sampling and Recruitment, 4.4 Population and Sample, and 4.5 Inclusion and Exclusion Criteria. | pp. 8–9, lines 175–201 |
| 7 | **Variables** | Clearly define all outcomes, exposures, predictors, potential confounders, and effect modifiers. Addressed in 4.2 Instrument, Validity, and Reliability (Outcomes; Governance predictors; Covariates). | pp. 7–8, lines 142–174; Supplementary File 2 |
| 8 | **Data sources/ measurement** | For each variable of interest, give sources of data and details of methods of assessment. Addressed in 4.2 Instrument, Validity, and Reliability and 4.7 Data Sources and Collection. | pp. 6–8, lines 129–174; p. 10, lines 208–211 |
| 9 | **Bias** | Describe any efforts to address potential sources of bias. Addressed in 4.2 (embedded attention check) and 6.1 Strengths and Limitations of the Work. | pp. 6–7, lines 130–137; pp. 18–19, lines 390–412 |
| 10 | **Study size** | Explain how the study size was arrived at. Addressed in 4.6 Sample Size and 4.4 Population and Sample. | pp. 9–10, lines 187–207 |
| 11 | **Quantitative variables** | Explain how quantitative variables were handled in the analyses, and which groupings were chosen and why. Addressed in 4.2 Instrument, Validity, and Reliability and 4.8 Data Analysis. | pp. 7–8, lines 153–174; pp. 10–11, lines 212–230 |
| 12 | **Statistical methods** | (a) Describe all statistical methods, including those used to control for confounding. (b) Describe methods used to examine subgroups and interactions. (c) Explain how missing data were addressed. (d) If applicable, describe analytical methods taking account of sampling strategy. (e) Describe any sensitivity analyses. Addressed in 4.8 Data Analysis, 4.6 Sample Size (power sensitivity analysis), and 5.4 (cumulative-threshold sensitivity models). | pp. 9–11, lines 187–230; pp. 13–14, lines 290–304 |
|  | *Results* |  |  |
| 13 | **Participants** | (a) Report numbers of individuals at each stage of the study. (b) Give reasons for non-participation at each stage. (c) Consider use of a flow diagram. Addressed in 4.4 Population and Sample, 4.5 Inclusion and Exclusion Criteria, and 5.1 Subjects. | pp. 8–9, lines 175–201; Supplementary File 2 |
| 14 | **Descriptive data** | (a) Give characteristics of study participants and information on exposures and potential confounders. (b) Indicate the number of participants with missing data for each variable. Addressed in 5.1 Subjects and Table 1; the analytic sample had complete data on all modeled variables. | pp. 11–12, lines 239–255; Table 1, pp. 25–26 |
| 15 | **Outcome data** | Report numbers of outcome events or summary measures. Addressed in 5.2 Work-Related Use and Clinical-Use Frequency. | p. 12, lines 256–266; Supplementary File 2 |
| 16 | **Main results** | (a) Give unadjusted and, if applicable, confounder-adjusted estimates and their precision (e.g., 95% confidence interval). (b) Report category boundaries when continuous variables were categorized. (c) If relevant, translate relative risk into absolute risk. Addressed in 5.3 and 5.4 and Tables 2 and 3. | pp. 12–14, lines 268–304; Tables 2–3, pp. 27–28 |
| 17 | **Other analyses** | Report other analyses done, e.g., subgroups, interactions, and sensitivity analyses. Addressed in 4.6 Sample Size and 5.4 (cumulative-threshold models retaining age). | pp. 9–11, lines 202–230; pp. 13–14, lines 291–304 |
|  | *Discussion* |  |  |
| 18 | **Key results** | Summarize key results with reference to study objectives. Addressed in 6 Discussion (Principal findings). | p. 14, lines 305–313 |
| 19 | **Limitations** | Discuss limitations, taking into account sources of potential bias or imprecision, and the direction and magnitude of any potential bias. Addressed in 6.1 Strengths and Limitations of the Work. | pp. 18–19, lines 390–412 |
| 20 | **Interpretation** | Give a cautious overall interpretation considering objectives, limitations, multiplicity of analyses, results from similar studies, and other evidence. Addressed in 6 Discussion, 6.2 Recommendations for Further Research, and 7 Conclusion. | pp. 14–19, lines 305–430 |
| 21 | **Generalisability** | Discuss the generalisability (external validity) of the study results. Addressed in 6.1 Strengths and Limitations of the Work. | pp. 18–19, lines 390–412 |
|  | *Other information* |  |  |
| 22 | **Funding** | Give the source of funding and the role of the funders. Addressed on the Title Page (Funding Statement). | Separate title page, Funding Statement |

*An Explanation and Elaboration article discusses each checklist item and gives methodological background and published examples of transparent reporting. The STROBE checklist is best used in conjunction with this article, available on the STROBE website (https://www.strobe-statement.org).*
