# Supplementary material for: Workplace Policy Awareness, Training, and Supervisory Support as Correlates of Generative Artificial Intelligence Use Among Practicing Nurses
Source: Nurs Open. 2026 Nov 3;13(11):e71000. doi: 10.1002/nop2.71000 (PMC13731167; doi:10.1002/nop2.71000)
Supplement: Supplementary file 2 [file NOP2-13-e71000-s002.docx]

Supplementary File 2

Participant flow, analytic items, response options, coding, and routing documentation for the reviewed sample of 1,136.

Item wording and offered response options below were checked against the questionnaire supplied on October 17, 2026. The full instrument and study data remain available from the corresponding author upon reasonable request; they are not reproduced in full here.

# Participant flow

| **Step** | **n** |
| --- | --- |
| Completed exported submissions | 1239 |
| Excluded not licensed in the United States | 6 |
| Excluded not employed in a nursing role | 4 |
| Excluded no direct-care supervisory or support role | 51 |
| Excluded failed or unreached attention-quality gate | 34 |
| Eligible and quality-passed records | 1144 |
| Excluded declined consent | 8 |
| Final complete-case analytic sample | 1136 |

Exclusions are sequential in the order shown. All retained respondents provided consent, met the three eligibility screens, passed the recorded attention-quality gate, and had complete values for the modeled variables. The 34 quality-gate exclusions are not all demonstrable incorrect attention-check responses; some respondents did not reach the gate. No imputation, weighting, or post-stratification was used.

# Quality checks and outcome reference frames

For the analytic sample, survey duration had median 399 seconds, mean 569 seconds, 5th percentile approximately 198 seconds, 95th percentile approximately 1,192 seconds, and range 98–37,683 seconds. No additional duration-based exclusion is applied in the supplied analysis syntax. There were no duplicate response IDs or panel IDs in the reviewed analytic sample.

Q56 measures any work-related use during the previous 12 months. Q57 measures habitual clinical-task frequency with no bounded recall period and is not conditional on Q56 in the supplied questionnaire. Among respondents answering No to Q56, 129 reported some clinical use and 40 reported weekly or daily clinical use on Q57. These outcome proportions are not nested or directly comparable.

# Questionnaire version and routing limitation

The supplied questionnaire shows end-of-block rules for Q1 = No; Q3, Q4, or Q5 = No; Q7 = Other; Q10 = Other; and Q74 other than Strongly disagree. An end-of-block rule is not, by itself, proof of survey termination. It also shows Q75 only when Q59 = No. The Q10 = Other rule is not consistent with treating that response as an automatic exclusion: 62 respondents with Other nursing roles have complete model data in the reviewed sample and were retained. The exact historical survey-flow configuration cannot be established from the later questionnaire alone. Complete historical survey-flow reconstruction is not claimed.

# Consent response

Q1 follows the study information sheet and offers Yes (1) or No (2). Only Yes respondents are analyzed. The complete study information sheet is available with the full instrument upon request. Declining consent is an exclusion even when other survey items were completed.

# Analytic questionnaire and coding

## Q3

Q3 Are you currently licensed to practice as a nurse in the United States? Yes (1) No (2)

Analytic coding. Yes is required for eligibility; No or missing is excluded.

## Q4

Q4 Are you currently employed in a nursing role? Yes (1) No (2)

Analytic coding. Yes is required for eligibility; No or missing is excluded.

## Q5

Q5 Do you provide, supervise, or support direct patient care in any clinical setting? Yes (1) No (2)

Analytic coding. Yes is required for eligibility; No or missing is excluded.

## Q7

Q7 What is the highest level of nursing education you have completed? Diploma in Nursing (2) Associate Degree in Nursing (3) Bachelor of Science in Nursing (4) Master of Science in Nursing (5) Doctor of Nursing Practice (DNP) (6) Doctor of Philosophy (PhD) (9) Other (8) [free-text field]

Analytic coding. Analytic education: 1 = Diploma or Associate Degree; 2 = Bachelor of Science; 3 = Master of Science, DNP, or PhD. Other is not an analytic education category; no retained case selected it.

## Q9

Q9 How many years have you practiced as a nurse? Less than 1 year (1) 1-5 years (2) 6-10 years (3) 11-20 years (4) More than 20 years (5)

Analytic coding. Analytic experience: 1–5 in the displayed order; entered as a linear ordinal term. Also evaluated categorically in the previously reported sensitivity analysis.

## Q10

Q10 Which of the following best describes your current nursing role? Bedside (1) Charge RN (2) Advanced Practice Nurse (Nurse Practitioner, Clinical Nurse Specialist, Nurse Midwife, Certified Registered Nurse Anesthetist) (11) Nurse manager or administrator (3) Nurse Educator (4) Other (9) [free-text field]

Analytic coding. Nominal predictor with Bedside as the reference; Charge RN, Advanced Practice Nurse, manager/administrator, educator, and Other retained. The 62 complete Other-role respondents are included.

## Q51

Q51 Which of the following best describes your primary employment setting? Hospital (1) Outpatient or Ambulatory (2) Home Health or Hospice (3) School Nurse (7) Public Health Nurse (8) Surgical Center (9) Other (4) [free-text field]

Analytic coding. Nominal predictor: Hospital reference; Outpatient/Ambulatory; Home Health/Hospice; Other combines School Nurse, Public Health Nurse, Surgical Center, and Other.

## Q12

Q12 In which U.S. Census region do you primarily practice? Northeast (1) Midwest (2) South (3) West (4) U.S. territory (5)

Analytic coding. Nominal predictor: South reference; Midwest, Northeast, West, and U.S. territory retained.

## Q15

Q15 Please select your gender from the options below. Female (1) Male (2) Other (3) [free-text field]

Analytic coding. Female reference; Male indicator. No analytic respondent selected Other. Do not recode a future Other or missing response as Female.

## Q50

Q50 Please select your age from the options below 18 - 24 (10) 25 - 34 (11) 35 - 44 (12) 45 - 54 (13) 55 - 64 (14) 65 - 74 (15) 75 - and Older (16)

Analytic coding. Observed age bands 18–24 through 65–74 coded 1–6 and entered as a linear ordinal term. The offered 75-and-older option has no retained respondents.

## Q56

Q56 The next questions ask about general-purpose AI tools such as ChatGPT, Claude, or Gemini — tools that can generate text, answer questions, or assist with writing. These are different from the clinical AI tools asked about in the previous section. All responses in this section are completely anonymous. In the past 12 months, have you used a generative AI tool (such as ChatGPT, Claude, or Gemini) for a work-related task? No (1) Yes (2)

Analytic coding. Binary outcome: No = 0, Yes = 1. Reference period is the previous 12 months.

## Q57

Q57 How often do you use generative AI tools for clinical tasks? Never (1) Less than once a month (2) About once a month (3) About once a week (4) Daily or almost daily (5)

Analytic coding. Ordered outcome: Never = 1, less than monthly = 2, monthly = 3, weekly = 4, daily/almost daily = 5 in the saved analytic file. The supplied SPSS reconstruction uses 0–4; the category order and regression interpretation are equivalent. No bounded recall period; no Q56-dependent skip is shown.

## Q58

Q58 The following questions ask about AI-related policies, training, and support in your workplace. Does your workplace have a policy governing the use of AI tools in patient care? Yes (1) No (2) I do not know (3)

Analytic coding. Nominal predictor with No as the reference; Yes and I do not know are separate categories.

## Q59

Q59 Have you received formal training on how to use AI tools in your clinical setting? Yes (1) No (2)

Analytic coding. Formal-training receipt combines with Q75 to create the three-level predictor. Yes = Trained reference, regardless of the structurally unasked Q75.

## Q75

Q75 Would you be interested in receiving formal training on how to use AI in the clinical setting? Yes (1) No (2)

Analytic coding. Displayed only if Q59 = No. Q59 No + Q75 Yes = Untrained interested; Q59 No + Q75 No = Untrained not interested. Q75 is structurally missing for trained respondents and is not modeled alone.

## Q60

Q60 My supervisor or manager actively supports the use of AI tools in patient care. Strongly disagree (6) Somewhat disagree (7) Neither agree nor disagree (8) Somewhat agree (9) Strongly agree (10)

Analytic coding. Analytic support score: 1–5 from Strongly disagree to Strongly agree; the questionnaire export uses option codes 6–10. Entered as a linear term; also evaluated categorically.

## Q61

Q61 I feel adequately supported by my organization to use AI safely in my practice. Strongly disagree (6) Somewhat disagree (7) Neither agree nor disagree (8) Somewhat agree (9) Strongly agree (10)

Analytic coding. Analytic support score: 1–5 from Strongly disagree to Strongly agree; the questionnaire export uses option codes 6–10. Entered as a linear term; also evaluated categorically.

## Q74

Q74 This item is an attention check. Please select "Strongly disagree" for this item. Strongly disagree (6) Somewhat disagree (7) Neither agree nor disagree (8) Somewhat agree (9) Strongly agree (10)

Analytic coding. Required response: Strongly disagree. Retention required the recorded quality gate gc = 1 and consent, with eligibility and completeness checked.
